# Substrate Stiffness Controls Osteoblastic and Chondrocytic Differentiation of Mesenchymal Stem Cells without Exogenous Stimuli

**DOI:** 10.1371/journal.pone.0170312

**Published:** 2017-01-17

**Authors:** Rene Olivares-Navarrete, Erin M. Lee, Kathryn Smith, Sharon L. Hyzy, Maryam Doroudi, Joseph K. Williams, Ken Gall, Barbara D. Boyan, Zvi Schwartz

**Affiliations:** 1 Department of Biomedical Engineering, Virginia Commonwealth University, Richmond, Virginia, United States of America; 2 Wallace H. Coulter Department of Biomedical Engineering, Georgia Institute of Technology, Atlanta, Georgia, United States of America; 3 Medshape, Inc., Atlanta, Georgia, United States of America; 4 School of Biology, Georgia Institute of Technology, Atlanta, Georgia, United States of America; 5 Children’s Healthcare of Atlanta, Atlanta, Georgia, United States of America; 6 Department of Mechanical Engineering and Materials Science, Duke University, Durham, North Carolina, United States of America; 7 Department of Periodontics, University of Texas Health Science Center at San Antonio, San Antonio, Texas, United States of America; University of Massachusetts Medical School, UNITED STATES

## Abstract

Stem cell fate has been linked to the mechanical properties of their underlying substrate, affecting mechanoreceptors and ultimately leading to downstream biological response. Studies have used polymers to mimic the stiffness of extracellular matrix as well as of individual tissues and shown mesenchymal stem cells (MSCs) could be directed along specific lineages. In this study, we examined the role of stiffness in MSC differentiation to two closely related cell phenotypes: osteoblast and chondrocyte. We prepared four methyl acrylate/methyl methacrylate (MA/MMA) polymer surfaces with elastic moduli ranging from 0.1 MPa to 310 MPa by altering monomer concentration. MSCs were cultured in media without exogenous growth factors and their biological responses were compared to committed chondrocytes and osteoblasts. Both chondrogenic and osteogenic markers were elevated when MSCs were grown on substrates with stiffness <10 MPa. Like chondrocytes, MSCs on lower stiffness substrates showed elevated expression of ACAN, SOX9, and COL2 and proteoglycan content; COMP was elevated in MSCs but reduced in chondrocytes. Substrate stiffness altered levels of RUNX2 mRNA, alkaline phosphatase specific activity, osteocalcin, and osteoprotegerin in osteoblasts, decreasing levels on the least stiff substrate. Expression of integrin subunits α1, α2, α5, αv, β1, and β3 changed in a stiffness- and cell type-dependent manner. Silencing of integrin subunit beta 1 (ITGB1) in MSCs abolished both osteoblastic and chondrogenic differentiation in response to substrate stiffness. Our results suggest that substrate stiffness is an important mediator of osteoblastic and chondrogenic differentiation, and integrin β1 plays a pivotal role in this process.

## Introduction

Millions of medical devices are implanted in Americans every year. These devices have a wide array of mechanical, chemical, and morphological properties. In vivo, implant surface properties including roughness, chemistry, energy, and topography affect bone-to-implant contact [1–4]. In vitro studies suggest that this is in part by stimulating osteoblastic differentiation of mesenchymal stem cells (MSCs) during bone healing [5].

Several reports have shown that MSCs are sensitive to substrate properties, such as surface roughness, stiffness, chemistry, and energy, and differentiate along specific lineages in response to these cues [6–10]. Substrate material properties play a role in inducing MSC differentiation into osteoblasts [11–13], even in the absence of exogenous factors or media supplements frequently used to stimulate osteogenesis in cultures grown on tissue culture polystyrene (TCPS) [5]. The specific role of stiffness has been more difficult to determine. Efforts to recapitulate the mechanical properties of extracellular matrix have suggested that specific stiffness can contribute to stem cell fate [14,15], but whether osteoblast differentiation is mediated by specific stiffness is not clear. Many studies were performed on metal and polymer substrates with lower or higher moduli range than native moduli of bone where such biomaterials generally are placed. Moreover, few studies have examined whether the effects of stiffness and chemistry are unique to osteoblastic differentiation or if other mesenchymal lineage fates might be induced as well.

Cells use mechanoreceptors to detect substrate stiffness via a mechanism that involves integrin-dependent signaling [14]. We have shown that integrin expression in MSCs and osteoblasts is modulated by surface properties, with α5β1 being expressed on smooth titanium and titanium alloy substrates and α2β1 being expressed on microtextured surfaces. Whereas α5β1 is associated with attachment and proliferation [16], α2β1 signaling is required for osteoblast differentiation [17]. Integrin β1 has been shown to mediate effects of other material and environmental stimuli on cell response [18,19] and has been demonstrated to play a role in chondrogenic differentiation [20,21].

Many studies examining how these properties modulate differentiation of multipotent cells like MSCs have focused on a single lineage fate. Relatively little is known about how changes in the chemical and mechanical microenvironment of these cells might differentially modulate phenotypic expression along multiple lineages [14,22]. In vivo, MSCs reside in tissues of varying stiffness and participate in tissue regeneration with stiffness changing as the repair tissue matures. This suggests that cells at different states within a lineage may respond differentially as they commit to a specific fate. To begin to examine this, we developed a series of polymer substrates with varying stiffness but without major changes in surface chemistry [23]. We found that a relatively high stiffness of 850 MPa was able to induce maturation of osteoblast-like MG63 cells. In the present study, we took advantage of methacrylate/methylmethacrylate polymer networks in which stiffness could be controlled by varying the amount of monomer [24], to investigate how stiffness mediates MSC commitment to two related lineages, osteogenic and chondrogenic, and compared MSC responses to those of committed osteoblasts and chondrocytes.

## Materials and Methods

### Polymer synthesis

Polymer substrates with elastic moduli orders of magnitude apart were synthesized to examine the effects of stiffnesses in ranges beyond those reported in the current literature and with moduli relevant to clinical applications. To accomplish this, we varied the weight ratio of methyl acrylate (MA) and methyl methacrylate (MMA) crosslinked with 10% poly(ethylene glycol) dimethacrylate (PEGDMA) [24]. Copolymer solutions consisting of MA, MMA, and PEGDMA MW~750 were obtained from Sigma-Aldrich and used as received. The weight ratio of MA to MMA was varied while the crosslinking concentration of PEGDMA was held constant at 10 wt% to produce four copolymer networks (by wt. % of MA): 18MA, 29MA, 40MA, and 72MA. 0.5 wt.% 2,2-dimethoxy-2-phenylacetophenone (DMPA) was used as the photoinitiator (Sigma-Aldrich). Each solution was mixed manually in a glass vial and injected between two glass slides using a glass pipette. Slides were separated with two 1mm glass spacers. The samples were placed in a UV chamber (Model CL-1000L Ultraviolet Crosslinker; λ = 365nm; energy = 2000x100μJ/cm 2) for 30 minutes. Discs were laser-cut from the polymerized sheets to a diameter such that the disc of each composition would swell to fill the bottom of a well in a 24-well cell culture plate when incubated in cell culture media. All discs were post-cured in an oven at 90°C for 90 minutes and boiled in distilled water for 30 minutes to remove excess monomer. Finally, discs were sterilized by UV light (λ = 254nm) for 90 minutes.

### Mechanical testing

Tensile strain-to-failure tests to determine toughness and elastic modulus were performed on a universal testing machine (MTS Insight 2) using a 2kN load cell and a strain rate of 5%/s. ASTM D632 Type IV Dogbone samples were laser-cut with a 20 mm gauge length and 2.8 mm gauge width, and their edges were sanded to remove any laser-induced defects. Samples were soaked in phosphate buffered saline (PBS) for 24 hours prior to testing, removed from PBS, patted with a paper towel to remove excess PBS, and their dimensions measured using digital calipers. Following this, the samples were loaded in tensile grips, submerged in a PBS bath at 37°C, and held at 37°C for 10 min to allow for thermal equilibration.

Toughness was calculated as the area under the stress-strain curve in units of MJ/m^3^. Elastic modulus was determined by calculating the slope of the linear portion of the stress-strain curve (n = 4). Dynamic mechanical analysis (DMA) in tensile loading was used to determine the rubbery modulus of the networks corresponding to the degree of crosslinking (TA Q800 DMA, Newcastle, DE). Rectangular samples of 1 x 5 x 15 mm^3^ were laser cut from polymer sheets, and their edges were sanded to remove any defects from the laser. The samples were thermally equilibrated at -75°C for 2 minutes and then heated to 200°C at a rate of 5°C/minute. Testing was performed in cyclic strain control at 0.2% strain with a preload force of 0.001 N and a force track setting of 150%. The glass transition temperature (T_g_) was defined as the peak of the tan delta curve, and the rubbery modulus was measured as the storage modulus value taken 20°C beyond the lowest point in the rubbery plateau (n = 3).

### Surface characterization

Surface wettability was determined by performing contact angle measurements using the sessile drop method (Ramé-hart Model 250 goniometer, Mountain Lakes, NJ) (n = 3). FTIR-ATR spectra were obtained on discs using a Bruker Optics Tensor Spectrometer (Billerica, MA) with a KBr crystal. Ten scans were performed on each sample at a 1 Hz frequency, and peak wavenumbers were determined using OMNIC software (Thermo Electron Corporation, Madison, WI). Three spectra were obtained for three separate discs for each composition.

### Cell studies

Human MSCs and human osteoblasts (HOBs, Lonza) were obtained from Lonza (Walkersville, MO). Human auricular chondrocytes were isolated from pediatric ear cartilage obtained under an IRB-approved protocol at Children’s Hospital of Atlanta and Georgia Institute of Technology. Informed consent was from the guardian and was in written form. The chondrocytes were isolated as described previously [25], cultured to confluence, and stored at -80˚C until used for this study. Auricular chondrocytes were chosen for their applications in tissue engineering, including their ability to proliferate and maintain their phenotype in culture [26–30]. In addition, we were interested in the modulation of phenotype along closely related lineages. Accordingly, we used auricular chondrocytes rather than articular or growth plate chondrocytes, to better identify specific stiffness modulating differentiation to an osteoblast or chondrocyte lineage.

Cells in passage two were used for all studies. Expression of cartilage cell phenotype at this passage was verified by gene expression of SOX9, ACAN, COL2, and COMP (S1 Fig), as described in the following paragraph. We did not assess expression of mRNA for elastin, a marker of the auricular chondrocyte phenotype, as our intent was to examine the general properties of osteoblasts v. chondrocyte lineage commitment.

All cells were grown plated at a density of 10,000 cells/cm^2^ on copolymer surfaces and cultured in Dulbecco’s modified Eagle’s medium (Corning, Manassas, VA) supplemented with 10% fetal bovine serum (Life Technologies, Carlsbad, CA) and 1% penicillin-streptomycin (Life Technologies). Cells were fed with this medium for 24 hours post-plating and every other day. After 7 days of culture, cells were incubated with fresh medium for 12 hours. RNA was isolated (TRIzol, Life Technologies) and quantified using a NanoDrop spectrophotometer (Thermo Scientific, Waltham, MA). To create a cDNA template, 500 ng of RNA was reverse transcribed using a High Capacity Reverse Transcription cDNA kit (Life Technologies). To quantify expression of RUNX2 mRNA in MSCs and HOBs, cDNA was used for real-time PCR with gene-specific primers (S1 Table) using the StepOnePlus Real-time PCR System and *Power* SYBR*®* Green PCR Master Mix (Life Technologies). Fluorescence values were quantified as starting quantities using known dilutions of cells cultured on tissue culture polystyrene (TCPS). mRNA expression was normalized to glyceraldehyde 3-phosphate dehydrogenase. Total cell number and cellular alkaline phosphatase specific activity were measured in the cell lysate as previously described [31].

Secreted osteocalcin (OCN, Biomedical Technologies, Stoughton, MA) and osteoprotegerin (R&D Systems, Minneapolis, MN) were measured to determine osteogenic differentiation. Immunoassays were normalized to total cell number. Chondrogenic differentiation was determined by measuring the expression of mRNAs for aggrecan (ACAN), cartilage oligomeric matrix protein (COMP), and collagen type II (COL2) as described above. Cartilage matrix production was assessed using an Alcian blue assay (Sigma-Aldrich, St. Louis, MO) to measure sulfated glycosaminoglycans. In brief, cell layers were fixed with 10% neutral buffered formalin for 10 minutes at room temperature. Cells were washed twice with PBS, then incubated with 3% acetic acid for 10 minutes. Proteoglycans were stained with 1% Alcian blue in 3% acetic acid (pH 2.5) for 30 minutes at room temperature. Cell layers were washed twice, and Alcian blue was extracted with dimethyl sulfoxide. Absorbance was measured at 650 nm [32].

For all experiments, MSCs, HOBs, and chondrocytes were grown at the same time with the same culture media to limit variability. To visualize cell shape, MSCs, HOBs and chondrocytes were plated on copolymer surfaces at a density of 5,000 cells/cm^2^ and allowed to spread for 24 hours in culture medium as described. Cell layers were fixed in 4% paraformaldehyde for 20 minutes and permeabilized in 0.05% Triton X-100 in PBS for 5 minutes. To visualize F-actin, cells were incubated for 1 hour with Alexa Fluor 488-labeled phalloidin (Life Technologies). At the end of the incubation period, cells were washed with PBS and incubated with Hoechst 33342 (Invitrogen) for 10 minutes. Finally, cultures were washed with 0.05% Triton X-100 in PBS, mounted on glass coverslips with Fluoro-Gel with Tris buffer (Electron Microscopy Sciences, Hatfield, PA) and imaged (Zeiss LSM 510 Non-Linear Optics with META Multiphoton Excitation, Carl Zeiss Microscopy, Thornwood, NY).

To examine integrin expression, MSCs, HOBs, and chondrocytes were plated on copolymer surfaces at a density of 10,000 cells/cm^2^ on copolymer surfaces and cultured in the same culture medium as described above. Cells were fed 24 hours post-plating and every other day thereafter. After 7 days, cells were incubated with fresh media for 12 hours and gene expression for integrin subunits α1 (ITGA1), α2 (ITGA2), α5 (ITGA5), αv (ITGAV), β1 (ITGB1), and β3 (ITGB3) measured as described above.

Permanently silenced ITGB1 MSCs were generated to examine integrin-dependent MSC differentiation on surfaces of varying stiffness. MSCs were transduced with shRNA lentiviral transduction particles (SHCLNV_NM_002211, TRCN 0000029645, Mission®, Sigma-Aldrich) to silence ITGB1. MSCs plated at 20,000 cells/cm^2^ were cultured overnight. Cells were incubated with particles (multiplicity of infection 5.0) overnight. Transduced cells were selected with culture media containing 0.25 μg/ml puromycin, yielding cells with 85% knockdown of mRNA. Quantification of mRNA levels of SOX9 and RUNX2, cell number, alkaline phosphatase specific activity, and secreted OCN and OPG were performed as described above and compared to wild-type cells. In preliminary experiments, there was no difference found between wild-type cells and cells containing empty vectors.

### Statistics

Data are shown as mean +/- SEM of six independent cultures from a representative experiment. All experiments were repeated. Using ANOVA with post-hoc Bonferroni’s modification of Student’s t-test a value of P < 0.05 was considered statistically significant.

## Results

**Fig 1:**
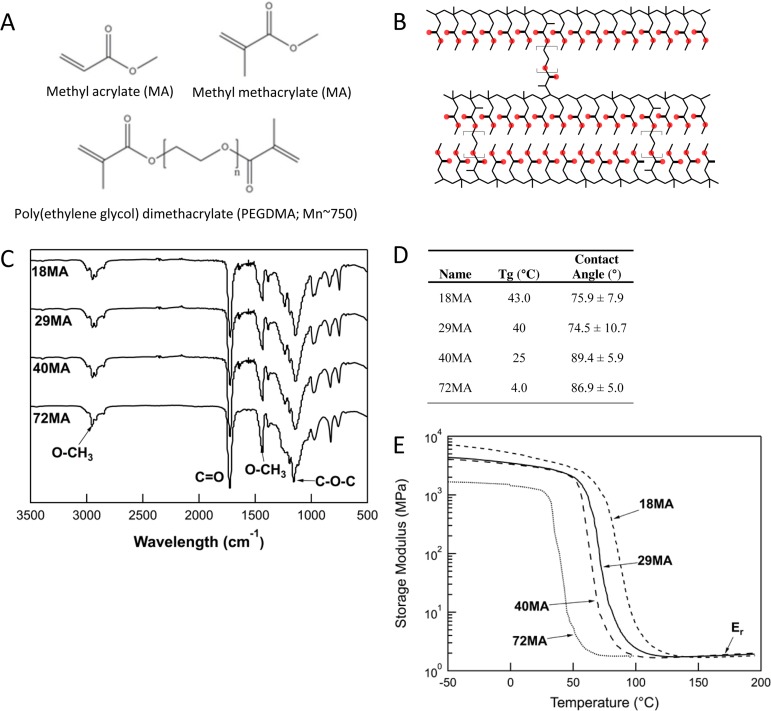
Characterization of MA-MMA networks. (A) Monomers used to create networks of increasing stiffness. (B) Example of crosslinked network. (C) Representative FTIR-ATR spectra for each network indicating similar surface chemistry for each. (D) Glass transition temperature (Tg) and contact angle of crosslinked networks. (E) DMA of crosslinked networks (n = 3).

**Fig 2:**
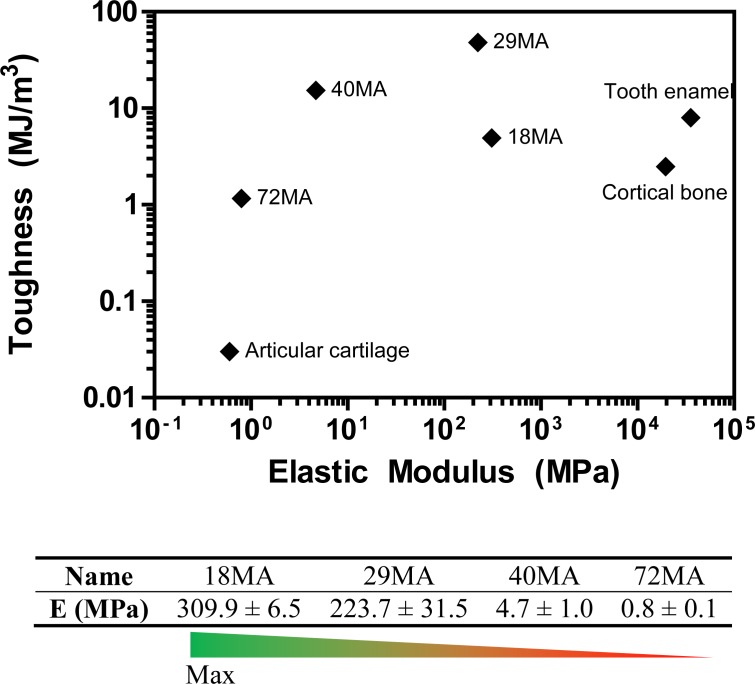
Toughness vs. elastic modulus for load-bearing biological tissues (green) and MA-MMA networks (black). Modified from data from [23].

The similar chemical makeup of the monomers (Fig 1A) yielded networks (Fig 1B) with similar surface chemistries as indicated by their FTIR-ATR spectra–the position of the major bonds (O-CH_3_, C = O, and C-O-C) did not shift between the different compositions (Fig 1C). Although these networks had similar surface energy as evidenced by similar contact angles (Fig 1D), their elastic moduli, measured in PBS at 37°C, spanned multiple orders of magnitude, (18MA>29MA>40MA>72MA) (Fig 1E). The toughness of the MA-MMA copolymers closely mimics the reported toughness of hard biological tissues including dentin and cortical bone (Fig 2).

**Fig 3:**
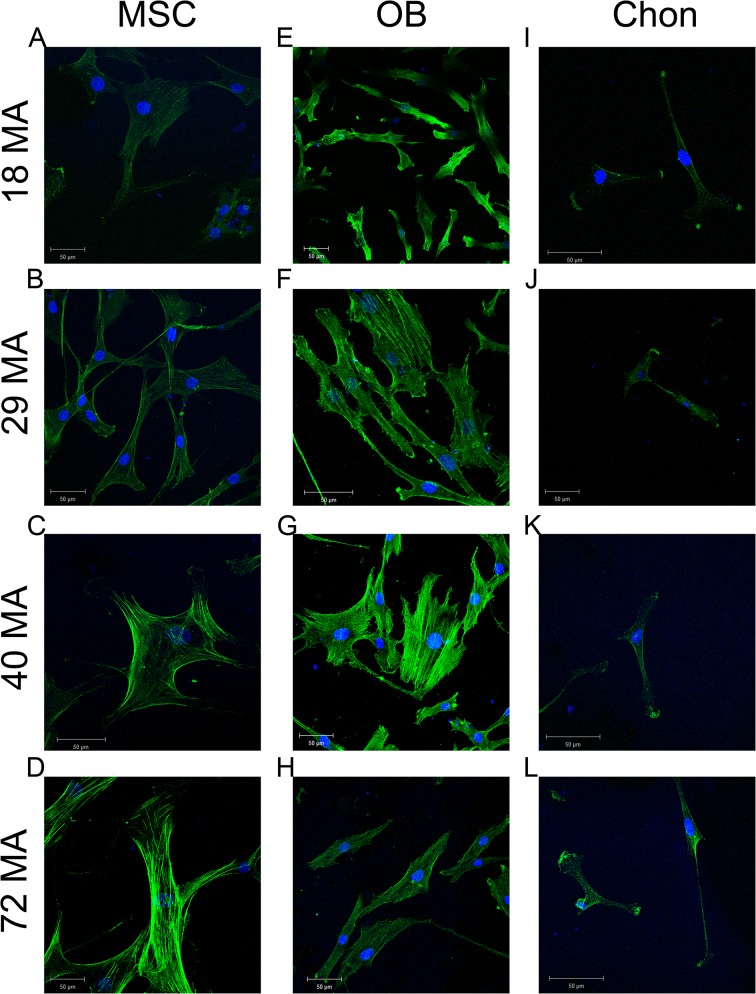
Cytoskeleton arrangement was altered by substrate stiffness. Representative staining of F-actin by phalloidin (green) and nuclei by DAPI (blue) in human MSCs (A-D), HOBs (E-H), and chondrocytes (I-L) cultured on surfaces of varying stiffness. (Scale bars: 100 μm for A,B,D; 50 μm for all others.)

Stiffness affected the structural organization of cytoskeletal filaments. MSCs grown on MA-MMA copolymer surfaces were longer on the less stiff surfaces, with multiple contact points on the 40MA surface (Fig 3A–3D). Unlike MSCs grown on surfaces with lower stiffness, F-actin appeared to be reduced in MSCs grown on 18MA surfaces (Fig 3A). HOBs were noticeably smaller on the 18MA surface (Fig 3E) and more spread out on the less stiff surfaces (Fig 3F–3H). Chondrocytes on 18MA, 29MA, and 40MA had similar morphology with long extensions and few points of contact (Fig 3I–3K), and began to spread wider on the least stiff surface (Fig 3L). However, there were no notable differences in the F-actin organization of HOBs or chondrocytes on the substrates examined (Fig 3E–3L).

**Fig 4:**
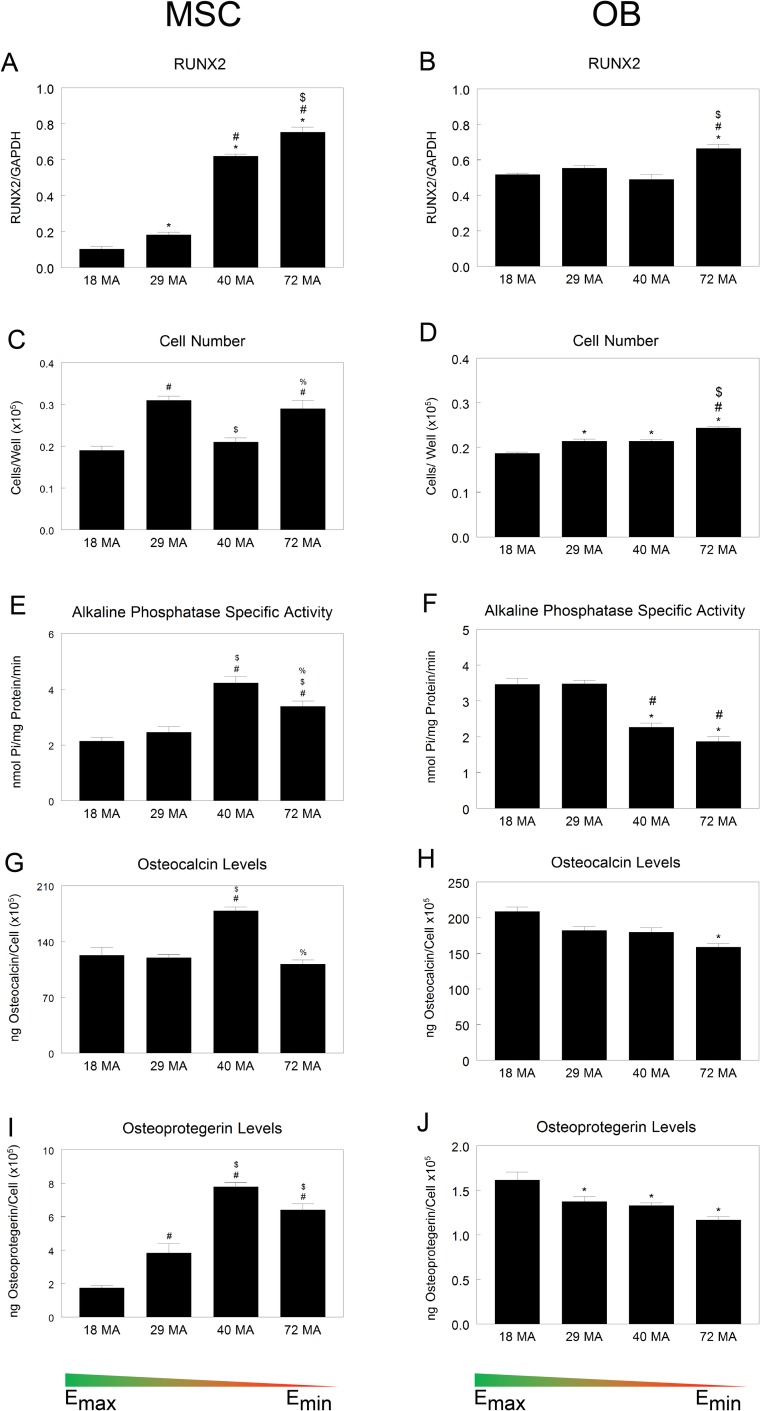
Osteoblastic differentiation on MA-MMA networks. (A-B) mRNA levels for osteoblast-specific marker RUNX2. (C-J) MSC and HOB response to substrate stiffness seen in cell number and osteogenic protein levels. *P < 0.05 vs. 18 MA, #*P* <0.05 vs. 29 MA, $*P* <0.05 vs. 40 MA.

We then compared the effects of stiffness on osteoblast phenotype in mMSCs and mature osteoblasts. MSCs and HOBs were grown without exogenous growth factors typically used to induce differentiation (see Materials and Methods). mRNA levels of transcription factor RUNX2 showed MSCs were more sensitive to stiffness than osteoblasts. RUNX2 mRNA in MSCs increased as substrate stiffness decreased, an effect not present in OBs; however, the highest RUNX2 levels were seen on 72MA surfaces in both cell types (Fig 4A and 4B). Cell number was significantly higher on the 29MA and 72MA surfaces for MSCs whereas in HOBs peak numbers were found on 72MA, followed by 29MA and 40MA (Fig 4C and 4D). Alkaline phosphatase specific activity, an early marker for cells in the osteoblast lineage, was greatest in MSCs grown on 40MA followed by 72MA while HOBs grown on these stiffnesses had lower alkaline phosphatase activity compared to 18MA (Fig 4E and 4*F*). mRNAs for matrix proteins osteocalcin and osteoprotegerin, associated with more mature osteoblasts, were similarly affected. Peak levels of these proteins occurred in MSCs grown on 40MA (Fig 4G and 4I). Conversely, there was a decrease in osteocalcin expression in HOBs grown on the least stiff substrates (Fig 4H), and osteoprotegerin mRNAs were lower in HOBs grown on all but 18MA (Fig 4J).

**Fig 5:**
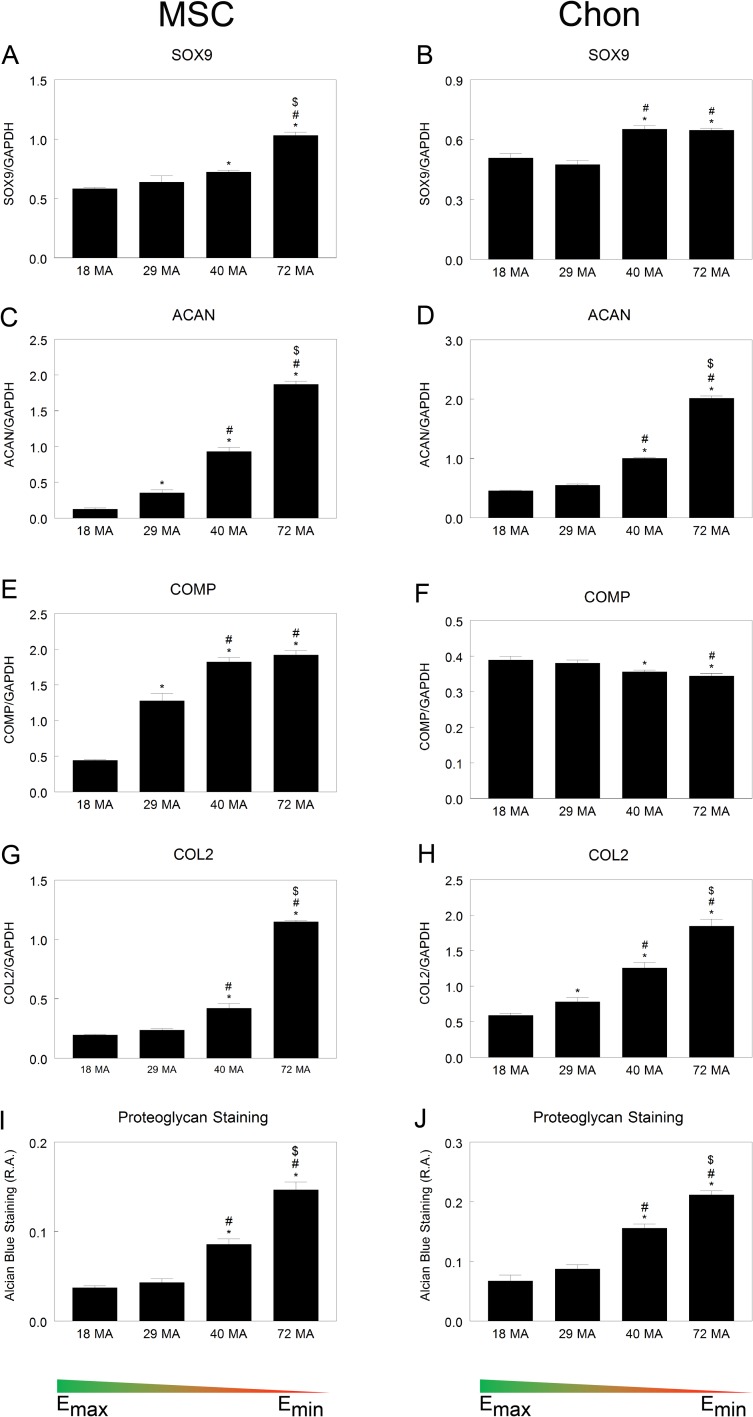
Chondrogenic differentiation on MA-MMA networks. Levels of chondrogenic mRNA (A-H) and quantification of proteoglycan staining in MSCs and chondrocytes (I-J) cultured on surfaces of varying stiffness. *P < 0.05 vs. 18 MA, #*P* <0.05 vs. 29 MA, $*P* <0.05 vs. 40 MA.

Chondrogenic differentiation of MSCs and chondrogenic markers in chondrocytes were responsive to substrate stiffness as well. MSCs and chondrocytes were grown for 7 days on the polymer surfaces without exogenous growth factors to induce differentiation (see Materials and Methods). SOX9, ACAN, COMP, and COL2 in MSCs increased as substrate stiffness decreased and were greatest on 72MA (Fig 5A, 5C, 5E and 5G), whereas levels of SOX9 were equally high in chondrocytes grown on 40MA and 72MA (Fig 5B). ACAN levels in chondrocytes mimicked MSCs for 40MA and 72MA (Fig 5C and 5D), but contrary to MSCs the level of COMP decreased on those same surfaces (Fig 5E and 5F). COL2 mRNA increased in chondrocytes as stiffness decreased, and the effect was similar to MSCs (Fig 5G and 5H). Alcian blue staining to detect sulfated glycosaminoglycans was similar for both MSCs and chondrocytes, with an increase in staining in cells grown on both 40MA and 72MA surfaces with the greatest intensity in cells grown on the least stiff surface (Fig 5I and 5J).

**Fig 6:**
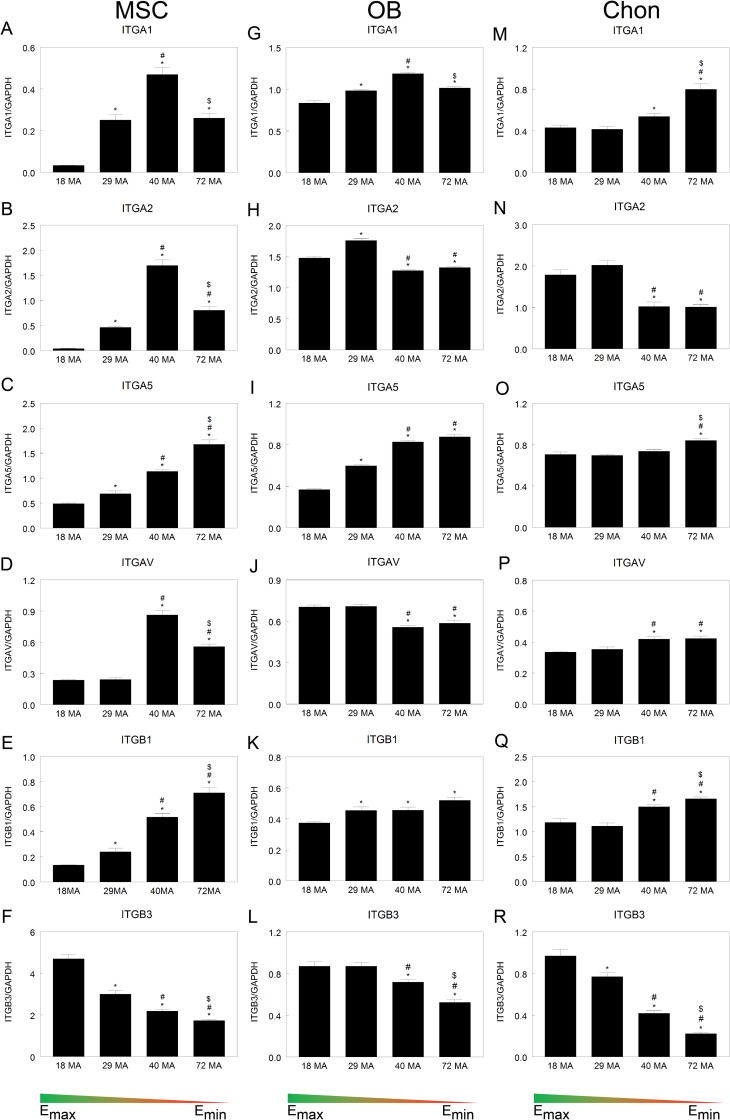
Integrin expression is stiffness- and cell-type- dependent. Comparison of integrin mRNA levels in MSCs, OBs, and chondrocytes cultured on surfaces of varying stiffness. *P < 0.05 vs. 18 MA, #*P* <0.05 vs. 29 MA, $*P* <0.05 vs. 40 MA.

Integrin expression was surface-dependent in all three cell types after seven days in culture. In MSCs, levels of integrin subunits ITGA1 and ITGA2 were higher on 29MA and 72MA substrates than on 18MA, with peak levels occurring in MSCs grown on the 40MA (4.7 MPa stiffness, Fig 6A and 6B). Surface stiffness altered mRNA levels of ITGA5 and ITGB1 in MSCs, with higher levels in MSCs grown on the least stiff surfaces (Fig 6C and 6E). Levels of IGTAV were significantly higher only on the 40MA and 72MA surfaces, peaking on the 40MA surfaces (Fig 6D). Conversely, ITGB3 increased as substrate stiffness increased (Fig 6F). In HOBs, ITGA1 levels were higher on all but the stiffest surface, peaking on the 40MA (Fig 6G), but levels of ITGA2 were highest in cells grown on 29MA and lower in those grown on the copolymers with lower stiffness (Fig 6H). HOBs grown on the surfaces with lower stiffness had higher levels of ITGA5 but lower levels of ITGAV (Fig 6I and 6J). The mRNA level for ITGB1 in HOBs was lowest on the stiffest surface (Fig 6K). A decrease in the levels of ITGB3 could be seen for HOBs grown on both 40MA and 72MA surfaces with the greatest decrease from those on the least stiff surface (Fig 6L). Finally, in chondrocytes, ITGA1 mRNAs increased in cells grown on the 40MA surface with higher levels in those grown on the 72MA surface (Fig 6M). The levels of ITGA2 were lower in chondrocytes grown on surfaces with lower stiffness (Fig 6N). Chondrocytes had similar levels of ITGA5 on all surfaces but were significantly higher from those grown on the least stiff surface (Fig 6O). The levels of both ITGAV and ITGB1 increased in chondrocytes grown on surfaces with lower stiffness (Fig 6P and 6Q) and, as in MSCs, a similar stiffness-dependent decrease in the level of ITGB3 was observed (Fig 6R).

**Fig 7:**
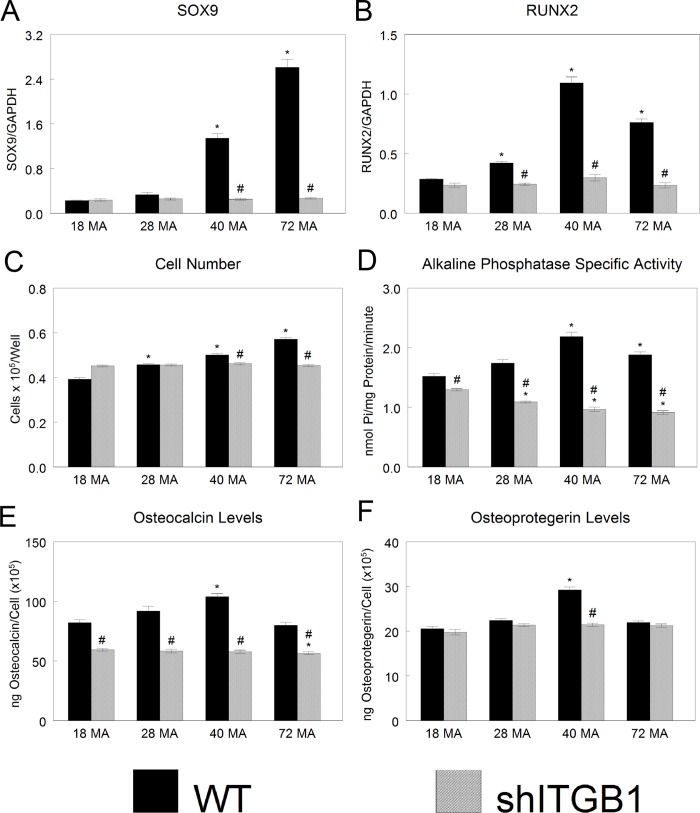
Integrin-dependent osteoblast differentiation. Levels of chondrogenic (A) and osteoblastic (B) mRNA. (C-F) Cell number and osteogenic protein levels in wild type human MSCs (WT) and silenced integrin β1 MSCs (shITGB1) cultured on surfaces of varying stiffness. *P < 0.05 vs. 18 MA, #*P* <0.05 vs. wild type.

Because MSCs tended to be the most sensitive to the varied stiffness, we wanted to determine how silencing integrin β1 (ITGB1) in these cells would modulate this response. Silencing ITGB1 abolished the stiffness-dependent expression of mRNA for transcription factors for chondrocytes (SOX9) and osteoblasts (RUNX2) (Fig 7A and 7B). Silencing ITGB1 also abolished the increase in cell number on decreasingly stiff surfaces seen in WT MSCs (Fig 7C). shITGB1-MSCs had lower alkaline phosphatase specific activity compared to their wild-type counterparts, and the levels decreased with decreasing stiffness (Fig 7D). Osteocalcin levels that were highest on 40MA in WT MSCs were lower in the silenced cells on all stiffness and lowest on 72MA surfaces (Fig 7E). Finally, the increase of osteoprotegerin in wild-type cells grown on 40MA was also abolished in shITGB1-MSCs (Fig 7F).

## Discussion

Multipotent stem cells from various sources have previously been shown to differentiate in response to varying topographies and stiffness [5,6,33–35]. In most cases, this differentiation has been enhanced with different induction media resulting in a very complex process that potentially masks effects of surface features, chemistry, or stiffness. In our study, we eliminated all but one of these variables, stiffness, in order to tease out its effects on MSC differentiation along two lineage fates: chondrogenic and osteogenic. Our results show that stiffness alone can direct differentiation and that different stiffness favors expression of a cartilage cell phenotype v. expression of an osteoblast phenotype, but no one stiffness produces an exclusive outcome.

We did not observe significant morphological changes in MSCs, HOBs, or chondrocytes on our polymer networks as others have demonstrated [6,36] although we did see some stiffness-dependent cytoskeletal arrangement. Significant changes in expression of differentiation markers do not necessarily correlate with outward changes in MSC morphology over the short time course of our study [37]. Similarly, we did not observe morphological changes in the chondrocyte and osteoblast cultures, although gene expression for differentiation markers was affected.

Gene expression in the cultures did vary with cell type and with substrate stiffness. MSCs exhibited increasing RUNX2 expression with decreasing stiffness, whereas expression in the committed HOB cells was less sensitive to substrate. Levels of this factor are correlated with osteoblastic differentiation of MSCs [38–40], suggesting that less stiff substrates induced osteoblastic differentiation. Whether this reflects *in vivo* differentiation on osteoclast resorbed bone surfaces, which are primarily collagen and non-collagenous proteins like osteopontin rather than stiffer fully mineralized bone [41,42], isn’t clear. The lower RUNX2 in HOBs on certain substrates suggests that the cells are less active osteoblasts [43,44] than on other substrates.

We had expected MSCs to behave more like HOBs on the stiffer surface and more like chondrocytes on the less stiff surfaces but MSC responses were observed on the less stiff polymers (40MA and 72MA) for both lineages, indicating the culture was a mixed population exhibiting both osteoblastic and chondrogenic markers: more osteoblastic markers on the slightly less soft surface and more chondrogenic markers on the softest surface. The observation that MSCs had the highest osteoblast response on the next to least stiff (40MA) networks and the highest chondrogenic response on the least stiff (72MA) networks is an indicator that there exists an optimal substrate stiffness to promote osteoblast differentiation and that it is not simply ‘the harder, the better.’

HOB expression levels of osteoblastic genes increased only on the stiffer surfaces; on the softer surfaces, they not only did not have this increase but also may have begun to dedifferentiate. This suggests that maintenance of an osteoblastic phenotype may require a stiffer microenvironment typical of mineralized bone. To achieve a stable osteoblast phenotype in MSCs grown on TCPS requires extensive time in culture to develop multi-layered nodules and requires the use of media supplements for as long as three weeks to support mineral formation within the nodules [45]. Our study did not examine the long-term effects of MSCs on the softer 72MA substrate to determine if stiffness alone would support stable osteoblastic differentiation and matrix mineralization. Continued culture on the softer substrate in the absence of media supplements could have an inhibitory effect on downstream osteoblastic differentiation.

The differentiated HOBs behaved differently than the MSCs on the varied stiffness, suggesting that as differentiation progresses, substrate stiffness continues to influence cell maturation. Metal and ceramic implants generally have moduli much higher than native bone [23,46–48]; any polymer scaffold or bone substitute must consider stiffness as a critical factor. Most published work examining cell response to substrate stiffness use polymers such as hydrogels, which have moduli orders of magnitude low er than 100 kPa, far below biological tissues such as dental tissue or cortical bone, which are at or near common implant sites [19,33,35,49–52].

In contrast, chondrocytes, which exist in a hydrogel environment *in vivo* [53] behaved very similarly to MSCs. It is important to note that this study was performed using auricular chondrocytes, which may have different responses to substrate stiffness than growth plate or articular chondrocytes. Several reports have demonstrated that auricular chondrocytes proliferate well in culture, maintain their phenotype, and are suitable for tissue-engineered constructs [26–29]. Moreover, other reports have demonstrated that they are able to heal defects in articular cartilage to a similar extent as articular chondrocytes [30]. Given these applications in tissue engineering, we chose to use them as a cell source for this experiment.

Our results indicate that differential expression of integrins in response to surface stiffness plays a crucial role in determining cell response, and that integrin signaling controls MSC differentiation. Because integrins are a cell’s primary response to substrate stiffness due to ligand binding [54–56], and change according to differentiation [57], it follows that depending on a cell’s phenotype it would have more or less sensitivity to substrate stiffness. Expression of ITGA1, ITGA2, and ITGA5 in particular in MSCs was much more sensitive to stiffness than in either osteoblasts or chondrocytes. Others have reported a similar increase in ITGA5 in murine fibroblasts though no substrates stiffer than 55 kPa were examined [54]. Sanz-Ramos *et al*. examined integrin expression in rat chondrocytes on surfaces of 2–20 Pa stiffness under normoxia and hypoxia conditions and saw a decrease in ITGA2 and ITGAV, but no differences in ITGA1, ITGB1, or ITGB3 under normoxia [56], whereas we did. The softness of the substrates examined could account for the difference in our data compared to theirs, as changes in ITGB3 expression were not seen until MSCs were grown on stiffer substrates. The silencing of ITGB1 completely abolished this sensitivity at one week, faster than the 2–3 weeks reported previously [55]. The abolition of the SOX9 response in MSCs by ITGB1 silencing is likely due to cellular stiffness and diffusion changes, as others have seen increased activation of ITGB1 on softer substrates [18,58].

## Conclusion

Our results show that stiffness can direct the fate of MSCs and suggest that over a very small range, induce bone or cartilage formation–or both, such as our 40MA networks, which showed an enhancement of bone *and* cartilage markers. Once cells commit to an osteoblast lineage, stiffness has an entirely different effect, suggesting that softer substrates could halt further osteoblast maturation. We were able to enhance chondrocyte markers in mature chondrocytes while the same networks inhibited osteoblast markers in mature osteoblasts. In addition we show that multiple integrins and in particular integrin β1 play a vital role in MSC sensitivity to stiffness. Understanding the importance of this mechanical property unlocks its useful potential for exploitation to control cell fate.

## Supporting Information

S1 Fig Expression of cartilage cell phenotype. Levels of chondrogenic mRNA cultured on TCPS for 7 days. #*P* < 0.05 vs. MSCs.
(TIF)

S1 Table Primer sequences used for Real-time PCR analysis of gene expression. (DOC)
