# Supplementary material for: Substrate Stiffness Controls Osteoblastic and Chondrocytic Differentiation of Mesenchymal Stem Cells without Exogenous Stimuli
Source: PLoS One. 2017 Jan 17;12(1):e0170312. doi: 10.1371/journal.pone.0170312 (PMC5240960; doi:10.1371/journal.pone.0170312)
Supplement: S1 Table — (DOC) [file pone.0170312.s002.doc]

**SUPPLEMENTAL INFORMATION**

**
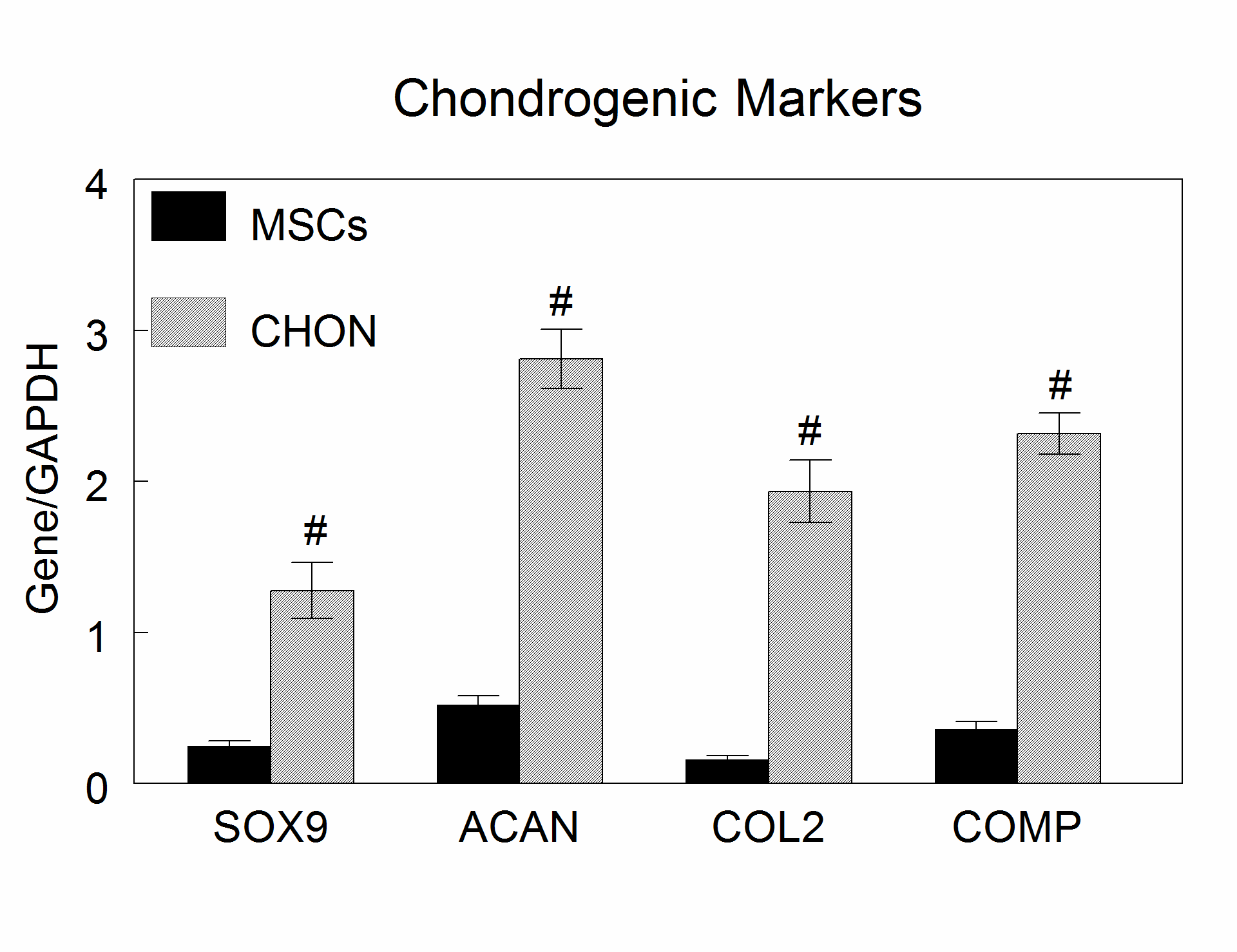
**

| **Gene** | **Primer Sequence** | |
| --- | --- | --- |
| ACAN | F | TCA GCG GTT CCT TCT CCA G |
|  | R | GCA GTT GTC TCC TCT TCT ACG |
| COL2A1 | Qiagen QuantiTect Primer Assay QT00049518 | |
| COMP | F | CCT GCG TTC TTC TGC TCA C |
|  | R | GCG TCA CAC TCC ATC ACC |
| GAPDH | F | GCT CTC CAG AAC ATC ATC C |
|  | R | TGC TTC ACC ACC TTC TTG |
| ITGA1 | F | CACTCGTAAATGCCAAGAAAAG |
|  | R | TAGAACCCAACACAAAGATGC |
| ITGA2 | F | ACT GTT CAA GGA GGA GAC |
|  | R | GGT CAA AGG CTT GTT TAG G |
| ITGA5 | F | ATC TGT GTG CCT GAC CTG |
|  | R | AAG TTC CCT GGG TGT CTG |
| ITGAV | F | GTTGCTACTGGCTGTTTTGG |
|  | R | CTGCTCCCTTTCTTGTTCTTC |
| ITGB1 | F | ATT ACT CAG ATC CAA CCA C |
|  | R | TCC TCC TCA TTT CAT TCA TC |
| ITGB3 | F | AAT GCC ACC TGC CTC AAC |
|  | R | GCT CAC CGT GTC TCC AAT C |
| OCN | F | GTG ACG AGT TGG CTG ACC |
|  | R | TGG AGA GGA GCA GAA CTG G |
| RUNX2 | F | GTC TCA CTG CCT CTC ACT TG |
|  | R | CAC ACA TCT CCT CCC TTC TG |
| SOX9 | F | TAG CCT CCC TCA CTC CAA GA |
|  | R | GGT TCGT TGG AGC TTT CCT TA |
